# Targeting Allosteric Site of PCSK9 Enzyme for the Identification of Small Molecule Inhibitors: An In Silico Drug Repurposing Study

**DOI:** 10.3390/biomedicines12020286

**Published:** 2024-01-26

**Authors:** Nitin Bharat Charbe, Flavia C. Zacconi, Venkata Krishna Kowthavarapu, Churni Gupta, Sushesh Srivatsa Palakurthi, Rajendran Satheeshkumar, Deepak K. Lokwani, Murtaza M. Tambuwala, Srinath Palakurthi

**Affiliations:** 1 Center for Pharmacometrics and Systems Pharmacology, Department of Pharmaceutics (Lake Nona), University of Florida, Orlando, FL 32827, USA; kowthavarapu.v@ufl.edu (V.K.K.); churnibidisha@ufl.edu (C.G.); 2 Facultad de Química y de Farmacia, Pontificia Universidad Católica de Chile, Santiago 8320000, Chile; fzacconi@uc.cl; 3 Institute for Biological and Medical Engineering, Schools of Engineering, Medicine and Biological Sciences, Pontificia Universidad Católica de Chile, Santiago 8320000, Chile; 4 Department of Pharmaceutical Sciences, Rangel College of Pharmacy, Texas A&M University, Kingsville, TX 78363, USA; spalakurthi@tamu.edu (S.S.P.); satheeshvad@gmail.com (R.S.); palakurthi@tamu.edu (S.P.); 5 Department of Pharmaceutical Chemistry, Rajarshi Shahu College of Pharmacy, Buldana 443001, India; dklokwani@gmail.com; 6 Lincoln Medical School, University of Lincoln, Brayford Pool Campus, Lincoln LN6 7TS, UK

**Keywords:** PCSK9, atherosclerotic cardiovascular disease, low-density lipoprotein, docking

## Abstract

The primary cause of atherosclerotic cardiovascular disease (ASCVD) is elevated levels of low-density lipoprotein cholesterol (LDL-C). Proprotein convertase subtilisin/kexin type 9 (PCSK9) plays a crucial role in this process by binding to the LDL receptor (LDL-R) domain, leading to reduced influx of LDL-C and decreased LDL-R cell surface presentation on hepatocytes, resulting higher circulating levels of LDL-C. As a consequence, PCSK9 has been identified as a crucial target for drug development against dyslipidemia and hypercholesterolemia, aiming to lower plasma LDL-C levels. This research endeavors to identify promising inhibitory candidates that target the allosteric site of PCSK9 through an in silico approach. To start with, the FDA-approved Drug Library from Selleckchem was selected and virtually screened by docking studies using Glide extra-precision (XP) docking mode and Smina software (Version 1.1.2). Subsequently, rescoring of 100 drug compounds showing good average docking scores were performed using Gnina software (Version 1.0) to generate CNN Score and CNN binding affinity. Among the drug compounds, amikacin, bestatin, and natamycin were found to exhibit higher docking scores and CNN affinities against the PCSK9 enzyme. Molecular dynamics simulations further confirmed that these drug molecules established the stable protein–ligand complexes when compared to the apo structure of PCSK9 and the complex with the co-crystallized ligand structure. Moreover, the MM-GBSA calculations revealed binding free energy values ranging from −84.22 to −76.39 kcal/mol, which were found comparable to those obtained for the co-crystallized ligand structure. In conclusion, these identified drug molecules have the potential to serve as inhibitors PCSK9 enzyme and these finding could pave the way for the development of new PCSK9 inhibitory drugs in future in vitro research.

## 1. Introduction

Atherosclerotic cardiovascular disease (ASCVD) is one of the leading causes of illnesses and deaths around the world [1]. Atherosclerotic disease has multiple causes; however, dyslipidemia and hypercholesterolemia are a major independent modifiable risk factor [2,3]. Dyslipidemia is characterized by increasing levels of cholesterol and triglycerides, particularly low-density lipoprotein cholesterol (LDL-C), as well as a decrease in high-density lipoprotein cholesterol (HDL-C) [4]. Similarly, the elevated level of LDL-C was also found in people with hypercholesterolemia, which can be treated with a variety of cholesterol-lowering drugs, among which statins are the most effective conventional drugs [5]. However, statins are associated with numerous adverse effects, such as myalgia, memory loss, type 2 diabetes, and many more, which are promoting the search for new cholesterol lowering medications [3]. To circumvent the limitations of cholesterol medication, researchers are working on developing proprotein converse subtilisin/kexin type 9 (PCSK9) inhibitors by employing a variety of strategies such as anti-sense oligonucleotides (ASOs), siRNA, monoclonal antibodies (mAbs), and peptide inhibitors. Among the distinct approaches, PCSK9 mABs [6] and siRNA inhibitors [7,8] have attracted a lot of interest from the most successful pharmaceutical companies in the world. Due to the significant incidence of dyslipidemia, PCSK9 inhibition has also emerged as a prominent focus of translational medicine research [9].

PCSK9 inhibitors, also referred to as PCKS9i, are highly potent medications for reducing LDL-C levels, significantly enhancing our capacity to address lingering cardiovascular risks. Normally, LDL binds to LDL receptors, facilitating LDL metabolism and the subsequent recycling of LDL receptors to the liver surface. Conversely, when PCSK-9 binds to LDL receptors, it triggers degradation. Consequently, LDL receptors cannot be recycled, leading to elevated LDL levels. Through the inhibition of PCSK9, a greater quantity of LDL receptors remains accessible on the liver’s surface, resulting in a decrease in blood cholesterol levels [8].

PCSK9 mAbs, Evolocumab, Alirocumab, and Inclisiran, were approved for clinical usage shortly after their discovery and have since been extensively utilized not only for treating familial hypercholesterolemia (FH) but also for secondary prevention of atherosclerotic cardiovascular disease (ASCVD) [10].

To further improve the therapeutic outcome and investigate the new approach, research continues to study the long-term safety and efficacy of PCSK9 inhibitors, as well as their role in specific patient populations. This includes exploring their use in combination with other cholesterol-lowering medications (Table 1). As of now, the field is dynamic, with ongoing clinical trials and studies providing new insights into the role of PCSK9 in cardiovascular health and potential therapeutic interventions (Table 1).

**Figure 1:**
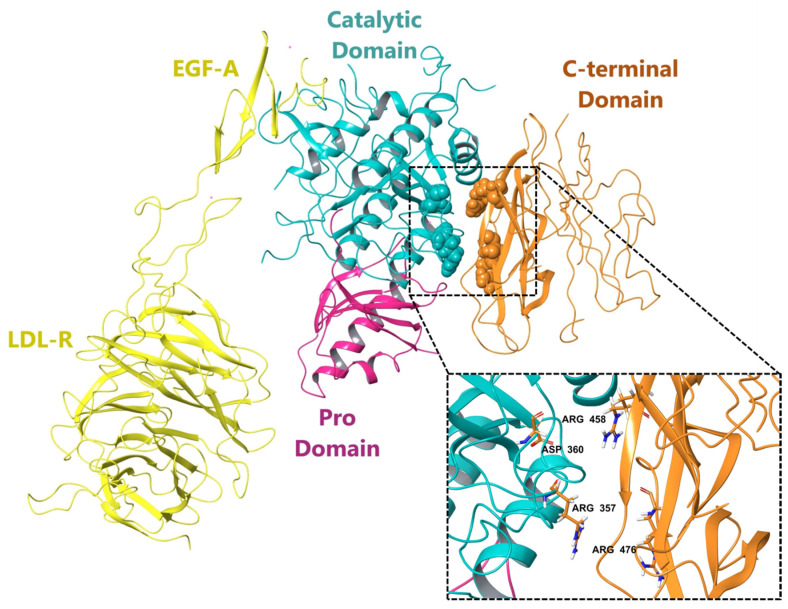
3D structure of the PCSK9-LDL-R complex (PDB ID: 3P5B) showing binding of EGF-A of LDL-R to the catalytic site of PCSK9 and key amino acid residues at the allosteric site of PCSK9 surface involved in binding to small molecules.

Furthermore, X-ray crystallographic structure and molecular dynamics studies have provided valuable insights into the interaction between PCSK-9 and the epidermal growth factor-like repeat-A (EGF-A) domain of the LDL receptor (LDL-R) [27,28]. It has been observed that PCSK-9 via its catalytic domain binds strongly to EGF-A, impairing the recycling of LDL-R to the cell surface. As a result, LDL-R undergoes lysosomal degradation under the influence of PCSK-9 protease activity, leading to elevated levels of LDL-R in the bloodstream [29,30,31,32]. Further, the analysis of the binding interface between PCSK-9 and EGF-A has emphasized the challenges in identifying small-molecule ligands for PCSK9 [28]. As reported, both proteins possess flat, featureless surfaces that primarily engage in hydrophobic interactions across a substantial area (Figure 1). Moreover, they form an antiparallel β-sheet structure [33], which adds further complexity to the interaction with small molecules [30]. Significantly, a distinctive allosteric binding site has been discovered between the catalytic and C-terminal domains of PCSK-9. This pocket holds promising potential for ligand binding and is positioned in proximity to various mutation sites that have been linked to both gain and loss of function. Furthermore, the interactions between certain amino acids, namely Arg357 and Asp360 in the catalytic domain, along with Arg458 and Arg476 in the C-terminal domains, at the allosteric site, have been identified as critical for stabilizing compounds within the allosteric site of PCSK-9.

Nevertheless, computer-aided drug design (CADD) plays a crucial role in identifying suitable binding sites within a target and facilitating the development of drugs that influence these binding sites. It also offers a promising way for drug repurposing, which involves discovering innovative treatment options at a low cost and with high efficiency. By repurposing drugs, we can explore their potential to target mutations or malfunctioning receptors and proteins, opening up exciting possibilities for novel therapies. In this study, we employed a structure-based virtual screening (SBVS) methodology to screen a library of FDA-approved drugs [34]. Our aim was to predict new inhibitory activity of these previously approved drugs against the PCSK9 enzyme. Additionally, we sought to elucidate the binding site of the PCSK9 enzyme and identify the amino acid residues crucial for drug binding. Through our comprehensive analysis, we aimed to uncover valuable insights that could pave the way for the development of potential PCSK9 inhibitors.

## 2. Methodology

### 2.1. Molecular Docking Using Glide and Smina

To identify potent inhibitors for PCSK-9 among FDA-approved drugs, we downloaded a library of 2992 FDA-approved drug compounds from the Selleckchem chemical database (https://www.selleckchem.com/, accessed on 25 July 2023). The 3D structures of the downloaded compounds were prepared and optimized to obtain lower energy conformers using Ligprep v3.5.9 (Schrodinger, LLC., New York, NY, USA). We also downloaded the X-ray crystal structure coordinates of the PCSK-9 enzyme (PDB ID: 6U2P) from the RCSB PDB with a resolution of 2.04 Å. This structure of PCSK-9 was co-crystallized with a small molecule inhibitor that contained a tetrahydroisoquinoline scaffold connected with a thiazole ring and substituted aromatic acid. The structure provided insights into the multiple intermolecular interactions between the ligand and the active site residues [35]. After downloading the structure, we prepared it for docking using the “protein preparation wizard” in Maestro v10.3 (Schrodinger, LLC.). The bond orders and formal charges for hetero groups were corrected and hydrogen atoms were added to all atoms in the structure. Side chains that were not in close proximity to the binding cavity and did not participate in salt bridges were neutralized, and the termini were capped by adding N-acetyl (ACE) and N-methyl amide (NMA) residues. Subsequently, the structure was refined to optimize the hydrogen bond network using the OPLS_2005 force field. The minimization process was terminated either when the energy converged or when the RMSD reached a maximum cutoff of 0.30 Å. For the molecular docking studies, molecular docking tool Glide v6.8 with the extra precision (XP) docking mode was employed [36,37]. The 6497 conformers generated for 2992 compounds using Ligprep were docked onto the generated grid of the PCSK9 protein structure. From the generated conformers, top 510 conformers of 268 compounds with docking scores better than −7.5 were selected for further analysis. These selected conformers were subjected to additional docking using Smina molecular docking software [38,39]. Smina is a customized version of AutoDock Vina that offers better support for scoring function development and high-performance energy minimization [39]. The binding site in the enzyme was defined based on the coordinates of the co-crystal ligand from the PDB, extended by 4 Å in each dimension. All 510 conformers of the 268 compounds were docked into the defined active site of the enzyme using the default scoring function of Smina.

### 2.2. CNN Scoring Using GNINA

To determine the CNN scoring of the best conformer for each compound that exhibited a good docking score in both Glide and Smina docking software, we performed score-in-place molecular docking using Gnina molecular docking software. Gnina is a modified version of Smina and AutoDock Vina, specifically designed to incorporate convolutional neural networks (CNN) for scoring protein–ligand poses [38,39,40]. In this study, Gnina was utilized to predict the quality of binding poses and binding affinity of protein–ligand complexes obtained from both Glide and Smina docking. The docking pipeline of Gnina takes advantage of the enhanced scoring capabilities provided by Smina, allowing the utilization of CNNs as scoring functions. A CNN scoring function has the ability to automatically learn the essential features of protein–ligand interactions that are associated with binding. The scores generated by the CNN models serve as an indication of the confidence in the quality of the ligand conformation generated during the docking process. In a typical usage scenario, Gnina requires a receptor structure, a ligand structure, and a specification for the binding site on the receptor.

### 2.3. Molecular Dynamics (MD) Simulation

Molecular dynamics (MD) simulations were conducted using the Desmond software (Desmond Molecular Dynamics System, D. E. Shaw Research, New York, NY, USA, 2020; Maestro–Desmond Interoperability Tools, Schrödinger, New York, NY, USA, 2020) following a previously reported procedure [41]. In summary, the MD simulations were performed to analyze the thermodynamic behavior and molecular stability of the lowest energy docked conformations of amikacin, natamycin, and bestatin, as well as the crystal structure-bound ligand and the apo form of the enzyme. To prepare the ligand–protein complexes for MD simulations, a simple point charge water model was employed, and an orthorhombic box with dimensions of 10 Å was used to solvate the complexes. Subsequently, ion neutralization was performed and the systems were minimized until a simulation time of 1 ns was reached. Following the equilibration period of 1 ns, each system was subjected to MD runs lasting 100 ns. The simulations utilized the OPLS_2005 force field parameters. The temperature and pressure were maintained at 300 K and 1.01325 bar, respectively, using the isothermal–isobaric (NPT) ensemble. For Coulomb interactions, a cutoff radius of 9 Å was employed, and the smooth Particle Mesh Ewald method was utilized for long-range interactions. Trajectories of the MD simulations were saved at intervals of 100 ps, resulting in approximately 1000 frames for subsequent analysis. Desmond’s simulation interaction diagram option was utilized to generate detailed information such as protein and ligand root mean square deviation (RMSD), root mean square fluctuation (RMSF), and ligand interaction profiles from the simulation trajectories. The stability of the MD simulations was monitored by observing the RMSD of the ligand and protein atom positions over time.

### 2.4. MM-GBSA Analysis

The thermal_MMGBSA.py script from the Prime/Desmond module of the Schrodinger suite was used to perform post-simulation molecular mechanics with generalized Born and surface area (MM-GBSA) analysis [42]. Every tenth frame was taken from the 100 ns of simulated trajectory for binding free energy estimates of ligands in complex with the PCSK9 protein, averaging a total of 100 frames. The OPLS forcefield-based VSGB 2.0 solvation model, which integrates residue-dependent effects was used for the polar solvation term [43]. The nonpolar solvation terms, i.e., solvent-accessible surface area (SASA), was also incorporated along with the van der Waals interactions to estimate the binding free energy (ΔG_bind_) of complex.

The ΔG_bind_ represents one of the most used parameters to estimate a ligand–protein binding which was estimated by subtracting the complex free energy from the sum of the individual free energies of the protein and ligand:

ΔG_bind_ = G(C) − G(P) − G(L)

where ΔG_bind_ is the total binding free energy, G(C) is the binding energy of protein–ligand complex, G(L) is the binding energy of ligand, and G(P) is the binding energy of protein. The binding energy of the receptor and ligand is calculated by the prime energy, a molecular mechanics + implicit solvent energy function (kcals/mol).

## 3. Result and Discussion

### 3.1. Structure-Based Virtual Screening Using Docking Studies

**Figure 2:**
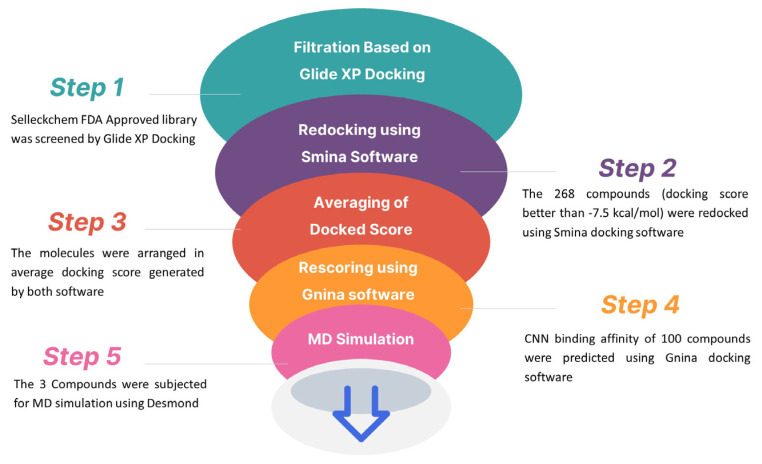
The virtual screening workflow illustrates the sequential steps used to identify potential PCSK9 inhibitors.

SBVS is a computational approach utilized to predict optimal ligand–target interactions and the formation of complexes [44]. SBVS enables the ranking of ligands based on their affinity to the target, with the most promising compounds appearing at the top of the list. This strategy involves selecting compounds from a database and categorizing them according to their affinity to the receptor site. In our study, we employed a structure-based in silico screening approach on a library of FDA-approved drugs consisting of 2992 compounds obtained from the Selleckchem chemical database, aiming to identify novel therapeutic candidates with good binding affinity against the PCSK9 enzyme. The screening process involved the docking of all FDA-approved drugs to the PCSK9 enzyme using the Glide extra-precision (XP) docking mode. From the initial screening, 268 compounds were selected based on a docking score better than −7.5 kcal/mol for further docking using Smina docking software (Version 1.1.2). Subsequently, the molecules were ranked based on their average docking scores obtained from both software and the poses of the first 100 compounds were further rescored and evaluated by CNN binding affinity against the PCSK9 protein using the Gnina docking software. (version 1.0) Due to the variation in docking scores generated by Glide, Smina, and Gnina for each ligand, we selected three molecules, namely amikacin, bestatin, and natamycin, based on their superior average docking scores and higher CNN affinities, for subsequent molecular dynamics (MD) simulation and post-MD simulation studies (Figure 2).

Prior to performing virtual screening of the FDA-approved library, the docking methodology was validated by removing the bound ligand from the protein structure (PDB id 6U2P) and redocking it into the active site of the enzyme. The RMSD value was calculated to assess the superimposition of the docked ligand with the co-crystallized ligand structure. The RMSD values were determined to be 0.601 Å and 0.424 Å for the Glide and Smina docking methodologies, respectively. These values below 2.0 Å indicate that both docking software generated correct poses for the co-crystallized ligand, validating the reliability of the docking process [45]. The average docking scores for the bound ligands, amikacin, natamycin, and bestatin, were found to be −8.90, −10.23, −9.98, and −9.78, respectively (Table 2). These compounds were further evaluated using Gnina docking software, which employs convolutional neural networks (CNNs) as a scoring function. The CNN model in Gnina provides predictions for both pose quality (CNNScore) and binding affinity (CNNaffinity). The CNNScore ranks the poses of the ligands, while the CNNaffinity predicts the affinity of each ligand in ‘pK’ units, where a pK value close to 6 indicates 1 μM affinity. The CNNScore of the bound ligands, amikacin, natamycin, and bestatin, were found to be 0.865, 0.58, 0.35, and 0.53, respectively, while their CNNaffinity values were 6.78, 5.45, 6.62, and 5.49, respectively.

**Figure 3:**
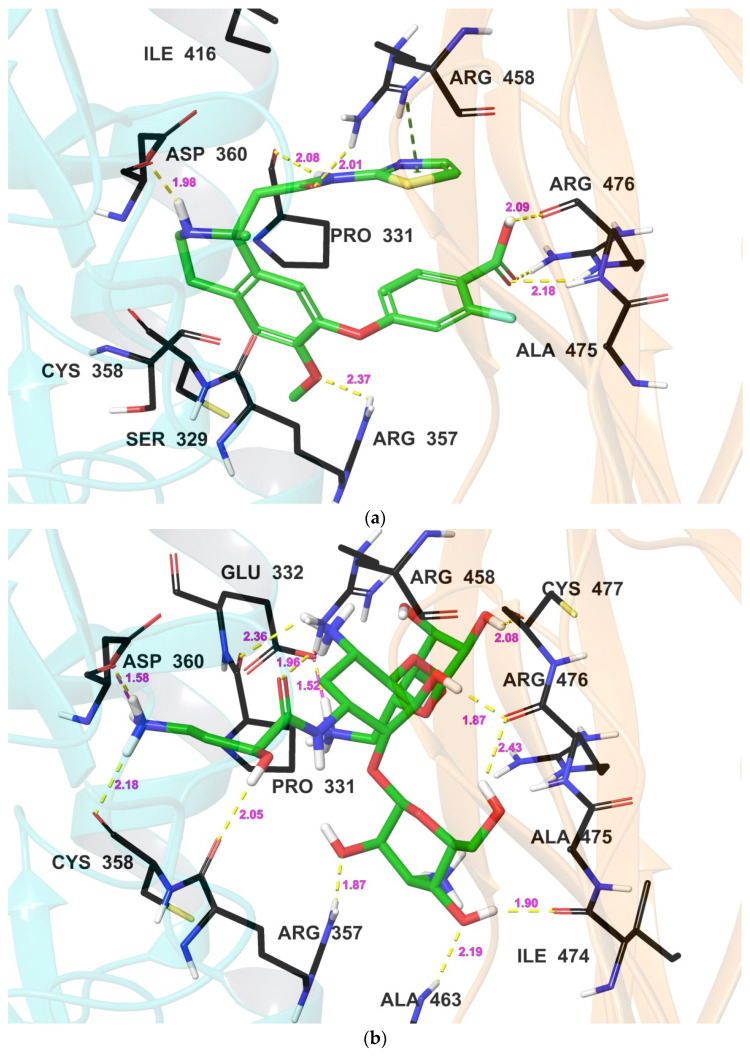

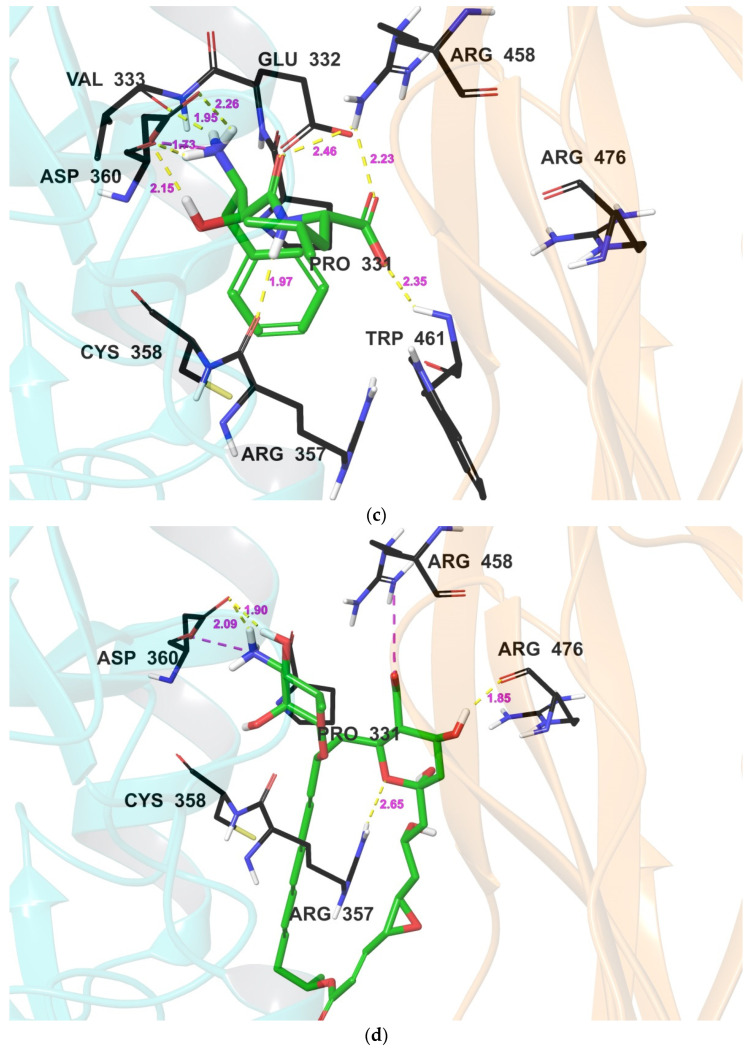
Binding pose of compounds (**a**) bound ligand, (**b**) amikacin, (**c**) bestatin, and (**d**) natamycin at the allosteric site of the PCSK9 enzyme showing the catalytic (cyan color) and C-terminal domain (orange color). H-bonds are denoted by yellow colored dotted lines with pink colored digits showing distance in Å. Magenta colored dotted lines indicate salt bridges. The green colored dotted line show the pi–cation interaction.

Figure 3 provides a comprehensive depiction of the binding poses of all compounds at the allosteric site of the PCSK9 enzyme. It was observed that all compounds interacted with at least one amino acid residue, including Arg357 and Asp360 in the catalytic domain, and Arg458 and Arg476 in the C-terminal domains, as reported in the literature for their interaction with small molecules [35]. All compounds exhibited specific binding at the allosteric site, encompassing both the catalytic and C-terminal domains, except for bestatin, which, due to its smaller structure, was shifted more towards the catalytic domain. In comparison to the crystal structure’s bound ligand, which showed H-bond interactions with amino acid residues Pro331, Arg357, Asp360, Arg458, and Arg476, the amikacin, with its higher number of hydroxyl groups, displayed hydrogen bond interactions not only with these amino acids but also with Glu332, Cys358, Ile474, and Cys477 (Table 3). This increased the stability of amikacin in the allosteric site of the PCSK9 enzyme. Similarly, natamycin exhibited H-bond interactions with all four crucial amino acid residues in the catalytic and C-terminal domains. However, as bestatin was shifted towards the catalytic site, it did not display hydrogen bonding interactions with Arg476, but it formed H-bonds with amino acid residues Glu332, Val333, Arg357, Asp360, Arg458, and Trp461.

Overall, molecular interactions of docked compounds and the PCSK9 enzyme indicate that the amikacin interacts with several amino acid residues of the PCSK9 enzyme through hydrogen bonds, including Pro331, Glu332, Arg357, Cys358, Asp360, Ala463, Ile474, Arg476, and Cys477. In addition, amikacin forms salt bridges with Glu332, Asp360, and Arg458. These interactions suggest that amikacin binds precisely between the catalytic and C-terminal domains of the allosteric site of the PCSK9 enzyme. Natamycin interacts with Arg357, Asp360, and Arg476 of the PCSK9 enzyme through hydrogen bonds. In addition, natamycin forms salt bridges with Asp360 and Arg458. These interactions suggest that natamycin is slightly shifted towards the catalytic site of the PCSK9 enzyme, as the direct H-bond interaction with Arg476 observed in the docking study was replaced by a water bridge. Bestatin was found to interact with several amino acid residues of the PCSK9 enzyme through hydrogen bonds, including Glu332, Val333, Arg357, Asp360, Arg458, and Trp461. In addition, bestatin forms salt bridges with Asp360. These interactions suggest that bestatin has a higher affinity towards the PCSK9 enzyme compared to the other compounds and the reference compound, as it demonstrated a better ΔG_bind_ value. The PDB bound ligand was found to interact with Arg357, Asp360, Arg476, and Arg458 of the PCSK9 enzyme through hydrogen bonds. In addition, it forms a pi-cation interaction with Arg458. These interactions suggest that the PDB bound ligand is positioned in the center of the allosteric site of the PCSK9 enzyme. Based on the molecular interactions, it can be concluded that all four compounds interact with key amino acid residues of the PCSK9 enzyme, including Asp360 and Arg458, which are known to be involved in the binding of PCSK9 to LDL-R. Targeting these residues could potentially inhibit the interaction between PCSK9 and LDL-R, leading to a reduction in LDL cholesterol levels.

### 3.2. MD Simulation Studies

**Figure 4:**
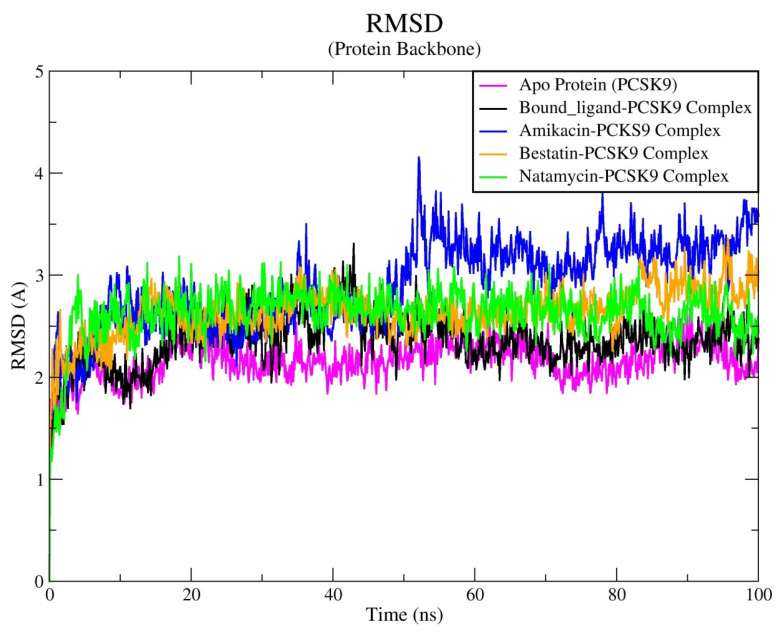
Time-dependent evaluation of RMSD (Å) for the PCSK9 protein alone and in complex with all docked ligands.

**Figure 5:**
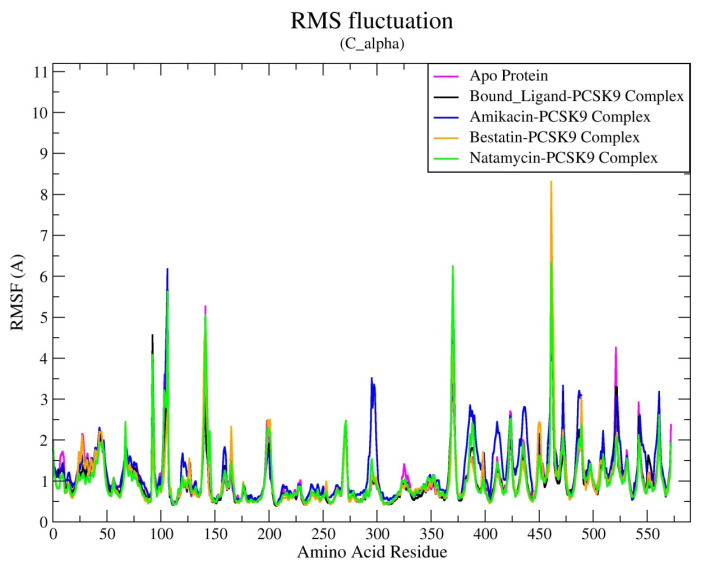
RMSF (Å) of Cα atoms of PSCK9 protein alone and in complex with all docked ligands after 100 ns MD simulations run.

**Figure 6:**
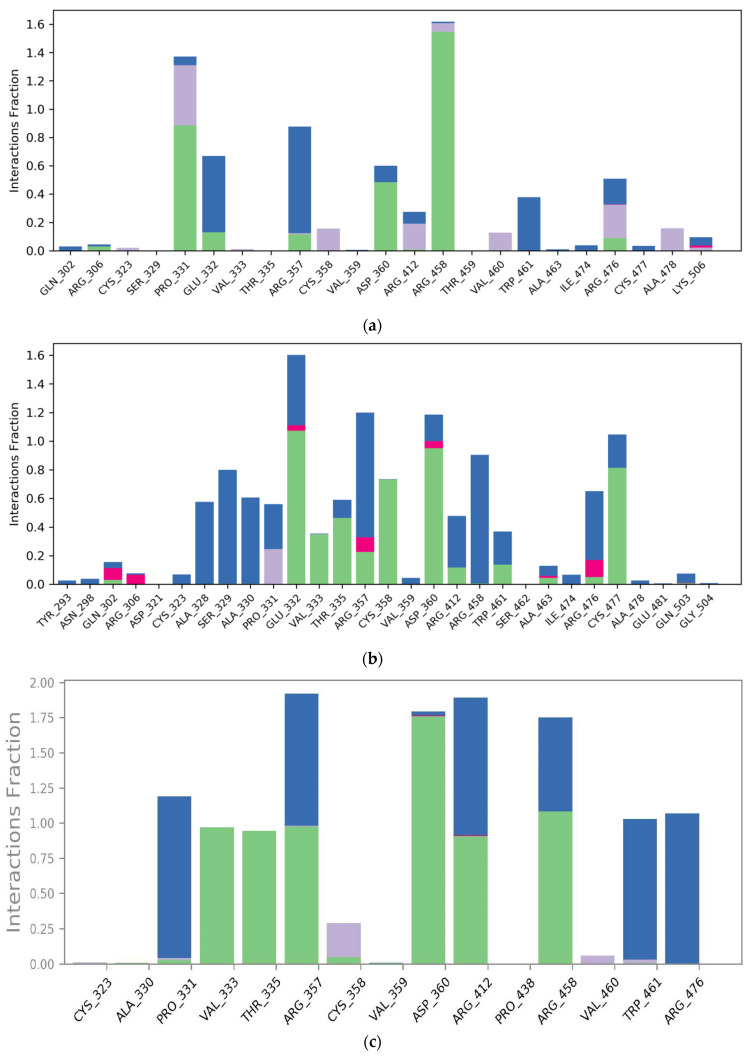

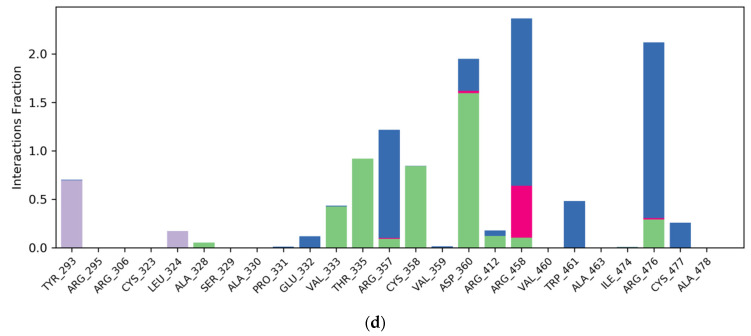
Protein–ligand contacts histogram obtained after 100 ns MD simulation studies. Figure shows the interactions percentage of amino acid residues for (**a**) bound ligand, (**b**) amikacin, (**c**) bestatin, and (**d**) natamycin. (Green colored bar—H-bonds, purple colored bar—hydrophobic interactions, blue colored bar—water bridges, and pink colored bar—ionic interactions).

**Figure 7:**
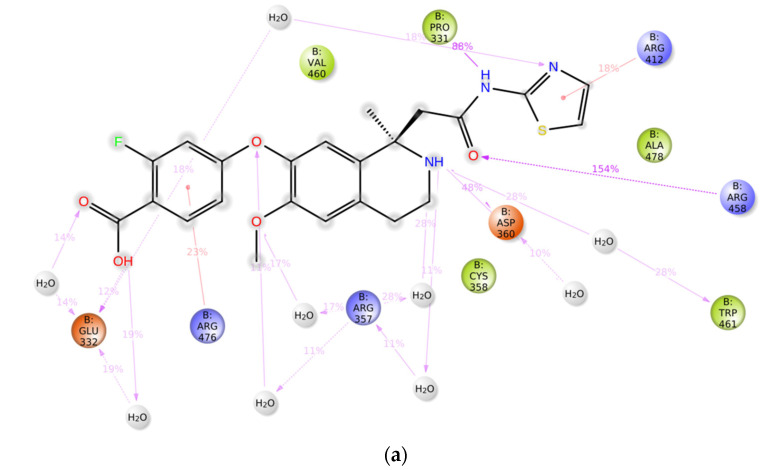

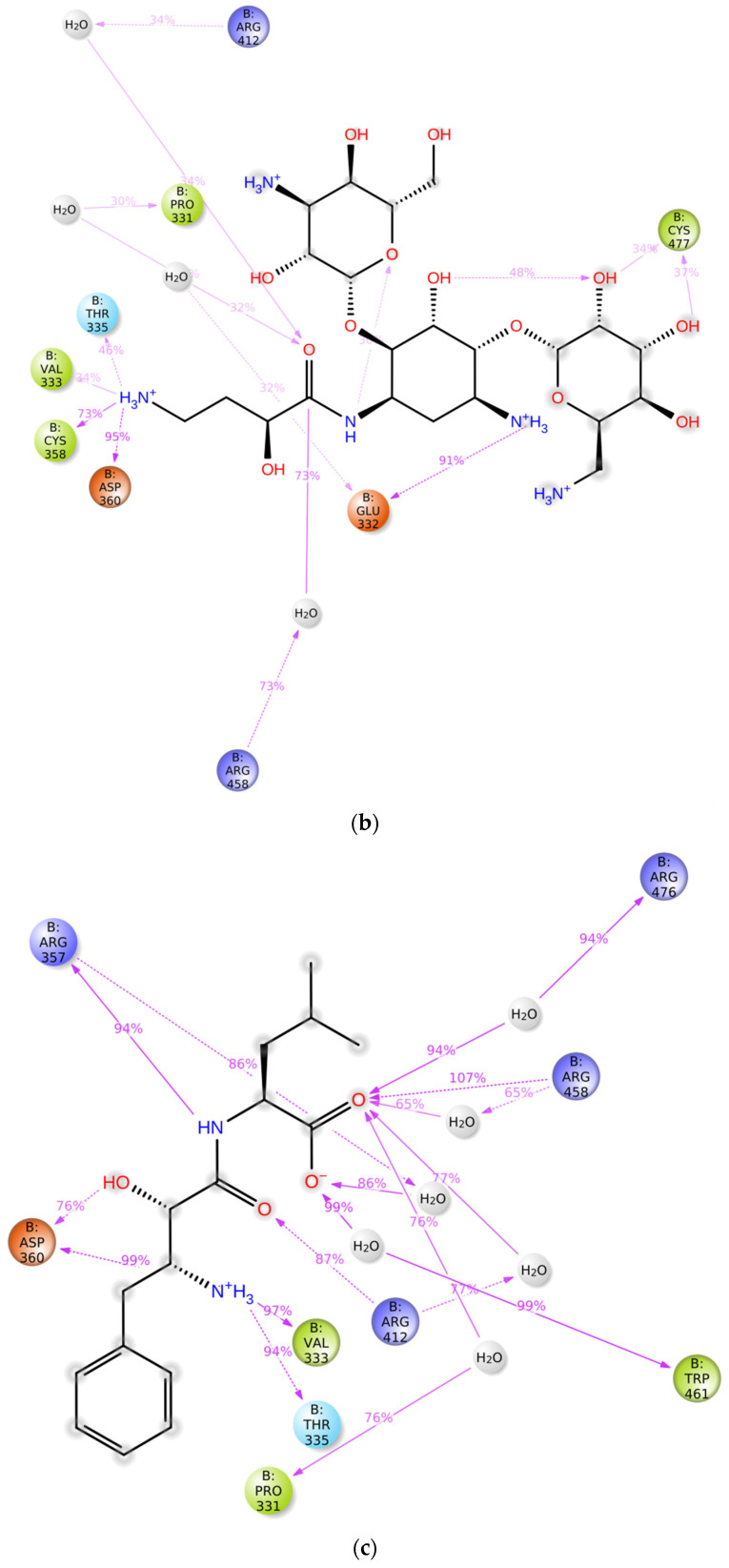

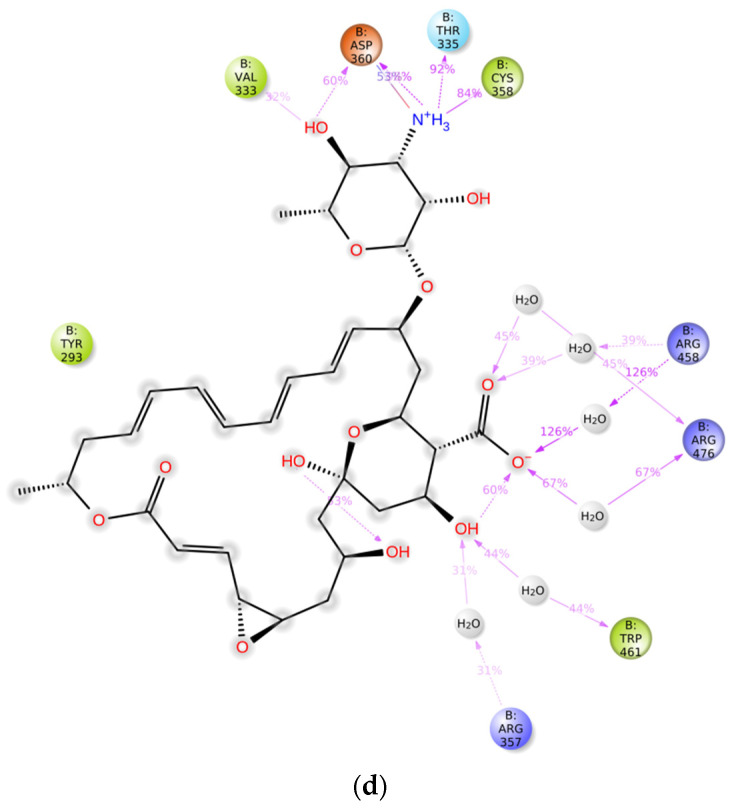
2D pose diagram showing protein–ligand contacts obtained after 100 ns MD simulation studies for (**a**) bound ligand, (**b**) amikacin, (**c**) bestatin, and (**d**) natamycin.

A molecular dynamics (MD) simulation study was conducted to investigate the behavior and stability of the PCSK9 enzyme alone and in combination with bound ligand, amikacin, natamycin, and bestatin. The MD simulations were carried out for 100 ns using the Desmond software (Desmond Molecular Dynamics System, D. E. Shaw Research, New York, NY, USA, 2020; Maestro–Desmond Interoperability Tools, Schrödinger, New York, NY, USA, 2020). Figure 4, Figure 5, Figure 6 and Figure 7 illustrate the key parameters utilized to assess the molecular stability of the docked compounds during the simulation. Various parameters, such as root mean square deviation (RMSD), root mean square fluctuation (RMSF), and contact mapping of the ligand–protein complexes, were analyzed to confirm the molecular behavior and stability of the compounds. These parameters were monitored over the entire 100 ns simulation duration to gain insights into the conformational changes that occur when the ligands are complexed with the PCSK9 protein.

The RMSD is a widely used metric to measure the average distance between atoms in different protein structures, typically focusing on the backbone atoms. It provides a measure of the overall displacement of atoms in one frame relative to a reference frame. The RMSD analysis of the apo protein backbone revealed that the PCSK9 protein without any ligand maintained a stable conformation throughout the MD simulation run, with RMSD values ranging between 2.0 and 2.5 Å. Upon the introduction of the bound ligand structures, the RMSD values increased initially, reaching up to 3.0 Å within the first 45 ns of the simulation, and then stabilized within the range of 2.0 to 2.5 Å. The complex backbone RMSD values for the amikacin–enzyme complex showed slight fluctuations up to first 50 ns MD run, then stabilizing between 3.0 and 3.75 Å. In contrast, both the natamycin–protein and bestatin–protein complexes exhibited greater stability, with backbone RMSD values ranging from 2.4 to 3.0 Å. Apart from the amikacin–protein complex, the other two complexes displayed a relatively stable molecular behavior of the protein with slight fluctuations. This indicates that the natamycin–protein and bestatin–protein complexes achieved a high level of equilibrium and dynamic stability during the MD simulation run (Figure 4). Furthermore, the RMSD values of the tested compound–protein complexes closely matched those of the reference bound ligand–protein complex as well as apo protein, suggesting comparable behavioral stability of the complexes.

Additionally, RMSF analysis was performed on the Cα atoms of the amino acid residues in all five systems (Figure 5). RMSF provides valuable insights into local changes along the protein chain by measuring the fluctuation of each residue throughout the entire simulation. Notably, apart from the loop regions, the Cα atoms in the active site of the enzyme exhibited lower atomic fluctuations, suggesting minimal conformational changes. The RMSF analysis also confirmed the stability of each amino acid within the ligand–protein complexes, indicating that all compounds formed stable complexes with the PCSK9 enzyme with minimal structural alterations. Overall, RMSF analysis showed that the compound–protein complexes closely matched those of the reference bound ligand–protein complex as well as apo protein, suggesting comparable behavioral stability of the complexes. This indicates that the natamycin–protein and bestatin–protein complexes achieved a high level of equilibrium and dynamic stability during the MD simulation run. It is concluded that all compounds interacted with both the catalytic and C-terminal domains of the PCSK9 enzyme through common amino acid residues, including Pro331, Glu332, Val333, Arg357, Cys358, Asp360, Arg458, Trp461, Arg476, and Cys477. It can be concluded that all four compounds form stable complexes with the PCSK9 enzyme with minimal structural alterations. The compounds interact with key amino acid residues of the PCSK9 enzyme, including Asp360 and Arg458, which are known to be involved in the binding of PCSK9 to LDL-R.

We conducted further analysis to investigate the formation and stability of hydrogen bonds under dynamic conditions. Figure 6 represents a comprehensive overview of the individual occupancies of H-bond interactions that occurred during MD simulation between ligands and proteins. Consistent with the docking studies, the protein–ligand contacts plot revealed that all compounds interacted with both the catalytic and C-terminal domains of the PCSK9 enzyme through common amino acid residues, including Pro331, Glu332, Val333, Arg357, Cys358, Asp360, Arg458, Trp461, Arg476, and Cys477. When considering direct H-bond interactions without involving water bridges, the bound -ligand structure showed interactions with amino acid residues Pro331, Asp360, and Arg458, with an interaction fraction of over 0.5, indicating that these interactions were present for more than 50% of the MD simulation. Similarly, amikacin exhibited direct H-bond interactions with amino acid residues Glu332, Thr335, Cys358, Asp360, and Cys477, with an interaction fraction of over 0.5, indicating its precise binding between the catalytic and C-terminal domains of the allosteric site of PCSK9 enzyme. In line with the docking study, bestatin also displayed direct H-bond interactions with amino acid residues Val333, Thr335, Arg357, Asp360, Arg412, and Arg458 throughout the MD simulation. However, bestatin also formed a water bridge with Arg476, which remained stable for more than 50% of the MD simulation, suggesting it’s positioning in the center of the allosteric site contrary to the docking result. Conversely, natamycin demonstrated direct H-bond interactions with amino acid residues Thr335, Cys358, and Asp360, and an ionic interaction with Arg458. This indicates a slight shift of natamycin towards the catalytic site, as the direct H-bond interaction with Arg476 observed in the docking study was replaced by a water bridge with an interaction fraction of over 0.5.

Figure 7 represents a 2D pose diagram illustrating the ligand–protein contacts after the MD simulation run. The figure provides a clear visualization of the percentage of interactions between specific ligands and various surrounding amino acid residues at the allosteric site of the PCSK9 enzyme during the 100 ns MD simulation. Notably, the amino acid residue Arg458 in the C-terminal domain was found to play a crucial role in the formation of H-bond interactions and/or water bridges, which remained stable for over 70% of the MD simulation with all four compounds. It was also observed that Arg458 consistently acted as a hydrogen bond donor in its interactions with the compounds, predominantly interacting with the oxygen of the carbonyl group. Furthermore, all of the compounds contributed to the stability of the ligand–protein complex by forming H-bond interactions or water bridges with the amino acid residue Asp360, which acted as a hydrogen bond acceptor and primarily interacted with amino groups.

Overall, the results of the MD simulations indicated that the ligand–protein docking complexes maintained conformational stability and exhibited consistent structural flexibility throughout the entire MD run.

### 3.3. Binding Free Energy Calculations of the Complexes Using MM-GBSA Analysis

The MM-GBSA analysis revealed a significant correlation between the experimental and predicted binding affinity through Gibbs free energy calculations. To further validate the binding affinity of the ligands against the protein, post-MD simulation MM-GBSA calculations were conducted. The ΔG_bind_, which estimates the binding free energy variation, was used as one of the key parameters for evaluating ligand–protein binding. The MM-GBSA ΔG_bind_ was calculated by comparing the energy difference between the bound and unbound states of the complexes. For the post-dynamic MM-GBSA analysis, 100 frames from the trajectories of each ligand–protein complex were selected at intervals of 10 ns. The binding free energy of these 100 systems in each simulation was computed using Prime software (version 4.0), and the mean values were reported (Figure 4). The calculated average ΔG_bind_ values ranged from −84.22 to −76.39 kcal/mol, indicating a high binding affinity of the compounds against the allosteric site of the PCSK9 enzyme. It is important to note that the MM-GBSA scoring function is optimized for predicting binding free energies in a congeneric series of molecules and, therefore, the absolute values calculated may not necessarily align with experimental binding affinities. However, the ranking of the ligands based on the calculated ΔG_bind_ is expected to reasonably correspond to the ranking based on experimental binding affinity, especially for congeneric series. The ΔG_bind_ values of all three drug compounds were observed near to bound ligand, which further indicates higher binding affinity against the PCSK9 enzyme. As ΔG_bind_ values represent approximate free energies of binding, a more negative value indicates stronger binding. Interestingly, all four systems exhibited negative ΔG_bind_ values, indicating strong stability of the systems. Additionally, bestatin demonstrated a better ΔG_bind_ value compared to the other compounds and the reference compound, suggesting a higher affinity towards the PCSK9 enzyme (Table 4).

The Prime MM-GBSA method utilizes the VSGB 2.0 solvation model, which incorporates the OPLS_2005 force field for both bonded and non-bonded terms, as well as a solvation term and several physics-based correction terms for hydrogen bonding, π-π interactions, self-contact interactions, and hydrophobic interactions. From the Table 5, it is apparent that the non-bonded terms, such as van der Waals (ΔG_vdW_, −68.91 to −59.47 kcal/mol), Coulomb (ΔG_Coul_, −60.67 to −11.16 kcal/mol), and lipophilic (ΔG_Lipo_, −23.85 to −18.93 kcal/mol) energy terms, play a major favorable role in binding ligands to the PCSK9 enzyme. The physics-based energy term, hydrogen bonding (ΔG_Hbond_, −4.82 to −2.83 kcal/mol), moderately supported the binding of the compounds. However, it was observed that the electrostatic solvation energy (ΔG_Solv_, 16.67 to 59.47 kcal/mol) strongly disfavored the binding. Additionally, the covalent binding (ΔG_Coval_, 3.96 to 6.86 kcal/mol) and π-π packing (ΔG_packing_, −0.43 to 1.57 kcal/mol) energy terms also moderately disfavored the binding of compounds to the enzyme. The high negative values of ΔG_Coul_ and ΔG_Lipo_ indicate that the allosteric site of PCSK9 is lined with both polar and nonpolar residues, and interaction with these residues through hydrogen bonding increases the affinity. Furthermore, the high negative values of ΔG_vdW_ suggest that the tested compounds were well embedded within the allosteric sites upon binding. Moreover, it is evident that compounds with balanced polar and nonpolar structural properties, along with a defined number of hydrogen bond acceptors, hydrogen bond donors, and hydrophobic groups, such as bestatin, exhibit good binding affinity for the PCSK9 enzyme compared to more polar compounds. Overall, MM-GBSA analysis revealed a significant correlation between the experimental and predicted binding affinity through Gibbs free energy calculations. The calculated average ΔG_bind_ values ranged from −84.22 to −76.39 kcal/mol, indicating a high binding affinity of the compounds against the allosteric site of the PCSK9 enzyme. Based on the results of the MM-GBSA analysis, it can be concluded that all four compounds have a high binding affinity towards the PCSK9 enzyme. These results also suggest that the compounds have the potential to inhibit the interaction between PCSK9 and LDL-R, leading to a reduction in LDL cholesterol levels.

Energy contributing to the MM-GBSA binding free energy (ΔG_bind_) for selected virtual hits in the allosteric site of the PCSK9 enzyme was calculated. All the four compounds exhibited negative ΔG_bind_ values, indicating strong stability of the systems. Bestatin demonstrated a better ΔG_bind_ value compared to the other compounds and the reference compound, suggesting a higher affinity towards the PCSK9 enzyme.

## 4. Conclusions

In conclusion, the allosteric binding site of the PCSK9 enzyme and the amino acid residues were found to be crucial for drug binding. Targeting this site could be a promising approach for drug development. The 3D structure of the PCSK9-LDL-R complex, which shows the binding of EGF-A of LDL-R to the catalytic site of PCSK9 and key amino acid residues at the allosteric site of the PCSK9 surface involved in binding to small molecules was used for the docking studies to set up molecular docking and virtual screening studies towards novel PCKS9i. Furthermore, varying degrees of PCSK9 inhibition offer a spectrum of effects on LDL receptor activity and circulating cholesterol levels. A low degree provides modest benefits, potentially necessitating combination therapies, with minimal safety concerns. A moderate degree yields significant reductions in LDL cholesterol, making it a viable standalone therapy, although heightened monitoring is warranted. Meanwhile, a high degree achieves maximal efficacy but introduces greater risks, demanding careful patient selection and vigilant surveillance for adverse effects.

In the present work, the X-ray crystallography and molecular dynamics studies have provided valuable insights into the interaction between PCSK9 and the EGF-A domain of the LDL-R. Interactions between y Arg357 and Asp360 in the catalytic domain, along with Arg458 and Arg476 in the C-terminal domains, at the allosteric site, have been identified as critical for stabilizing compounds within the allosteric site of PCSK9.

According to the obtained Glide score values, amikacin was found to have the lowest Glide score of −11.50, followed by natamycin and bestatin, both with a Glide score of −11.37. The PDB bound ligand has the highest Glide score of −7.417, indicating weaker binding affinity compared to the other three compounds. Amikacin had the strongest binding affinity to the PCSK9 enzyme among the four compounds tested.

The high negative values of ΔG_vdW_ suggest that the tested compounds were well embedded within the allosteric sites upon binding. Furthermore, the results suggest that compounds with balanced polar and nonpolar structural properties, along with a defined number of hydrogen bond acceptors, hydrogen bond donors, and hydrophobic groups, such as bestatin, exhibit good binding affinity for the PCSK9 enzyme compared to more polar compounds. Based on the results of the energy terms contributing to the MM-GBSA binding free energy, it can be concluded that bestatin has the highest binding affinity towards the PCSK9 enzyme among the tested compounds. The results suggest that bestatin has the good potential to inhibit the interaction between PCSK9 and LDL-R, leading to a reduction in LDL cholesterol levels.

Overall, we suggest that targeting the allosteric site of the PCSK9 enzyme could be a promising approach for drug development, as it could lead to the identification of small molecule inhibitors that could stabilize the enzyme and prevent it from binding to the LDL receptor. This could ultimately lead to the development of novel therapies for atherosclerosis and other related diseases. However, further studies are needed to evaluate the potential of all the compounds as a PCSK9 inhibitor, including in vitro and in vivo studies to validate the predicted binding affinities and assess its pharmacokinetic properties.

## Figures and Tables

**Table 1:** Summary of the key aspects of current research activities based on PCSK9.

Aspect	Research Focus
Therapeutic Target	PCSK9 inhibitors for lowering LDL cholesterol [11]
Drugs in Focus	Evolocumab, Alirocumab, and other monoclonal antibodies [12]
Drug Development	Refining inhibitors, exploring new delivery mechanisms
Genetic Variations	Studying PCSK9 mutations and their impact on cholesterol [13]
Gene Editing	Exploring gene-editing technologies for therapeutic use [14]
Safety and Efficacy	Investigating long-term safety and efficacy of PCSK9 inhibitors [15]
Combinatorial Approaches	Studying the use of PCSK9 inhibitors in combination with other medications [16]
Patient Populations	Exploring efficacy and safety in specific patient groups [17,18,19,20,21,22]
Ongoing Clinical Trials	Continuous assessment of PCSK9 inhibitors in various stages [23,24,25,26]

**Table 2:** Docking score of selected compounds against the PCSK9 enzyme.

Sr. No.	Compound Name	Glide Docking and Gnina Scoring			Smina Docking and Gnina Scoring			Average Docking Scores and Gnina Scoring		
		Glide Score	CNN Score	CNN Affinity	Smina Score	CNN Score	CNN Affinity	Docking Score	CNN Score	CNN Affinity
1	Amikacin	−11.50	0.22	3.89	−10.23	0.58	5.45	−10.87	0.40	4.67
2	Natamycin	−11.37	0.19	3.55	−9.98	0.35	6.62	−10.67	0.27	5.085
3	Bestatin	−11.37	0.44	6.20	−9.78	0.53	5.49	−10.57	0.485	5.845
4	PDB Bound Ligand	−7.417	0.76	6.69	−10.40	0.97	6.87	−8.90	0.865	6.78

**Table 3:** Molecular interactions of docked compounds and PCSK9 enzyme.

Sr. No.	Compound Name	H-bond Interactions	Other Interactions
1	Amikacin	Pro331, Glu332, Arg357, Cys358, Asp360, Ala463, Ile474, Arg476, Cys477	Salt Bridges (Glu332, Asp360, Arg458)
2	Natamycin	Arg357, Asp360, Arg476	Salt Bridges (Asp360, Arg458)
3	Bestatin	Glu332, Val333, Arg357, Asp360, Arg458, Trp461	Salt Bridges (Asp360)
4	PDB Bound Ligand	Arg357, Asp360, Arg476, Arg458,	Pi-cation interaction (Arg458)

**Table 4:** Calculation of MM-GBSA binding free energy (ΔG_bind_) for selected virtual hits in the allosteric site of the PCSK9 enzyme.

Compound	ΔG_bind_ (kcal/mol)	Complex Energy	Receptor Energy	Ligand Energy
Amikacin	−76.39	−17,582.18	−17,096.68	−409.10
Bestatin	−84.22	−17,523.80	−17,037.76	−401.82
Natamycin	−79.30	−17,752.07	−17,257.43	−415.33
Bound Ligand	−77.44	−17,648.45	−17,158.87	−412.12

**Table 5:** The energy terms contributing MM-GBSA binding free energy (ΔG_bind_) for selected virtual hits in the allosteric site of the PCSK9 enzyme.

Compound	ΔG_bind_	^a^ ΔG_Coul_	^b^ ΔG_Solv_	^c^ ΔG_Lipo_	^d^ ΔG_vdW_	^e^ ΔG_H-bond_	^f^ ΔG_Cov_	^g^ ΔG_packing_
Amikacin	−76.39	−24.15	30.62	−21.76	−62.02	−2.83	3.96	0.11
Bestatin	−84.22	−11.16	16.67	−23.85	−68.91	−2.89	6.74	−0.43
Natamycin	−79.30	−46.30	45.85	−20.82	−60.38	−4.82	6.86	0.74
Bound Ligand	−77.44	−60.67	59.47	−18.93	−59.47	−4.62	5.42	1.57

^a^ Coulomb energy (electrostatic energy), ^b^ Electrostatic (polar) solvation energy, ^c^ Lipophilic energy (non-polar solvation energy), ^d^ van der Waals energy, ^e^ Hydrogen-bonding energy, ^f^ Covalent binding energy, ^g^ π-π packing energy.

## Data Availability

Data are contained within the article.

## References

[1] Ouyang M., Li C., Hu D., Peng D., Yu B. (2023). Mechanisms of Unusual Response to Lipid-Lowering Therapy: PCSK9 Inhibition. Clin. Chim. Acta.

[2] Hageman S.H.J., McKay A.J., Ueda P., Gunn L.H., Jernberg T., Hagström E., Bhatt D.L., Steg P.G., Läll K., Mägi R. (2022). Estimation of Recurrent Atherosclerotic Cardiovascular Event Risk in Patients with Established Cardiovascular Disease: The Updated SMART2 Algorithm. Eur. Heart J..

[3] Makover M.E., Shapiro M.D., Toth P.P. (2022). There Is Urgent Need to Treat Atherosclerotic Cardiovascular Disease Risk Earlier, More Intensively, and with Greater Precision: A Review of Current Practice and Recommendations for Improved Effectiveness. Am. J. Prev. Cardiol..

[4] Puratchikody A., Irfan N., Balasubramaniyan S. (2019). Conceptual Design of Hybrid PCSK9 Lead Inhibitors against Coronary Artery Disease. Biocatal Agric. Biotechnol..

[5] Ballantyne C.M., Banach M., Mancini G.B.J., Lepor N.E., Hanselman J.C., Zhao X., Leiter L.A. (2018). Efficacy and Safety of Bempedoic Acid Added to Ezetimibe in Statin-Intolerant Patients with Hypercholesterolemia: A Randomized, Placebo-Controlled Study. Atherosclerosis.

[6] Dayar E., Pechanova O. (2022). Targeted Strategy in Lipid-Lowering Therapy. Biomedicines.

[7] Taylan C., Weber L.T. (2023). An Update on Lipid Apheresis for Familial Hypercholesterolemia. Pediatr. Nephrol..

[8] Päth G., Perakakis N., Mantzoros C.S., Seufert J. (2022). PCSK9 Inhibition and Cholesterol Homeostasis in Insulin Producing β-Cells. Lipids Health Dis..

[9] Safouris A., Magoufis G., Tsivgoulis G. (2021). Emerging Agents for the Treatment and Prevention of Stroke: Progress in Clinical Trials. Expert Opin. Investig. Drugs.

[10] Guedeney P., Giustino G., Sorrentino S., Claessen B.E., Camaj A., Kalkman D.N., Vogel B., Sartori S., De Rosa S., Baber U. (2022). Efficacy and Safety of Alirocumab and Evolocumab: A Systematic Review and Meta-Analysis of Randomized Controlled Trials. Eur. Heart J..

[11] Raal F.J., Stein E.A., Dufour R., Turner T., Civeira F., Burgess L., Langslet G., Scott R., Olsson A.G., Sullivan D. (2015). PCSK9 Inhibition with Evolocumab (AMG 145) in Heterozygous Familial Hypercholesterolaemia (RUTHERFORD-2): A Randomised, Double-Blind, Placebo-Controlled Trial. Lancet.

[12] Libby P., Tokgözoğlu L. (2022). Chasing LDL Cholesterol to the Bottom—PCSK9 in Perspective. Nat. Cardiovasc. Res..

[13] Kaddoura R., Orabi B., Salam A.M. (2020). Efficacy and Safety of PCSK9 Monoclonal Antibodies: An Evidence-Based Review and Update. J. Drug Assess..

[14] Meng F.H., Liu S., Xiao J., Zhou Y.X., Dong L.W., Li Y.F., Zhang Y.Q., Li W.H., Wang J.Q., Wang Y. (2023). New Loss-of-Function Mutations in PCSK9 Reduce Plasma LDL Cholesterol. Arterioscler. Thromb. Vasc. Biol..

[15] Ding Q., Strong A., Patel K.M., Ng S.L., Gosis B.S., Regan S.N., Cowan C.A., Rader D.J., Musunuru K. (2014). Permanent Alteration of PCSK9 with in Vivo CRISPR-Cas9 Genome Editing. Circ. Res..

[16] Igweonu-Nwakile E.O., Ali S., Paul S., Yakkali S., Teresa Selvin S., Thomas S., Bikeyeva V., Abdullah A., Radivojevic A., Abu Jad A.A. (2022). A Systematic Review on the Safety and Efficacy of PCSK9 Inhibitors in Lowering Cardiovascular Risks in Patients with Chronic Kidney Disease. Cureus.

[17] Wang X., Wen D., Chen Y., Ma L., You C. (2022). PCSK9 Inhibitors for Secondary Prevention in Patients with Cardiovascular Diseases: A Bayesian Network Meta-Analysis. Cardiovasc. Diabetol..

[18] Bradley C.K., Shrader P., Sanchez R.J., Peterson E.D., Navar A.M. (2019). The Patient Journey with PCSK9 Inhibitors in Community Practice. J. Clin. Lipidol..

[19] Kosmas C.E., Skavdis A., Sourlas A., Papakonstantinou E.J., Genao E.P., Uceta R.E., Guzman E. (2020). Safety and Tolerability of PCSK9 Inhibitors: Current Insights. Clin. Pharmacol..

[20] Vicente-Valor J., García-González X., Ibáñez-García S., Durán-García M.E., de Lorenzo-Pinto A., Rodríguez-González C., Méndez-Fernández I., Percovich-Hualpa J.C., Herranz-Alonso A., Sanjurjo-Sáez M. (2022). PCSK9 Inhibitors Revisited: Effectiveness and Safety of PCSK9 Inhibitors in a Real-Life Spanish Cohort. Biomed. Pharmacother..

[21] Chamberlain A.M., Gong Y., Shaw K.M.A., Bian J., Song W.L., Linton M.F., Fonseca V., Price-Haywood E., Guhl E., King J.B. (2019). PCSK9 Inhibitor Use in the Real World: Data from the National Patient-Centered Research Network. J. Am. Heart Assoc..

[22] Chng B.L.K., Heng W.M.P., Soon Y.M., Hon J.S., Lau Y.H., Tan R.S., Tan J.W.C. (2022). Safety, Adherence and Efficacy of PCSK9 Inhibitors: A Retrospective Real-World Study. Proc. Singap. Healthc..

[23] Arca M., Celant S., Olimpieri P.P., Colatrella A., Tomassini L., D’Erasmo L., Averna M., Zambon A., Catapano A.L., Russo P. (2023). Real-World Effectiveness of PCSK9 Inhibitors in Reducing LDL-C in Patients with Familial Hypercholesterolemia in Italy: A Retrospective Cohort Study Based on the AIFA Monitoring Registries. J. Am. Heart Assoc..

[24] Vikarunnessa S., Talloczy Z., Zang X., Pharma N., Maheux S.P., Lesogor A., Springer Cardiovascular F., Ray K.K., T Troquay R.P., J Visseren F.L. (2023). Long-Term Efficacy and Safety of Inclisiran in Patients with High Cardiovascular Risk and Elevated LDL Cholesterol (ORION-3): Results from the 4-Year Open-Label Extension of the ORION-1 Trial. Artic. Lancet Diabetes Endocrinol.

[25] Rallidis L.S., Skoumas I., Liberopoulos E.N., Vlachopoulos C., Kiouri E., Koutagiar I., Anastasiou G., Kosmas N., Elisaf M.S., Tousoulis D. (2020). PCSK9 Inhibitors in Clinical Practice: Novel Directions and New Experiences. Hell. J. Cardiol..

[26] Deedwania P., Murphy S.A., Scheen A., Badariene J., Pineda A.L., Honarpour N., Keech A.C., Sever P.S., Pedersen T.R., Sabatine M.S. (2021). Efficacy and Safety of PCSK9 Inhibition with Evolocumab in Reducing Cardiovascular Events in Patients with Metabolic Syndrome Receiving Statin Therapy: Secondary Analysis from the FOURIER Randomized Clinical Trial. JAMA Cardiol..

[27] Shapiro M.D., Tavori H., Fazio S. (2018). PCSK9: From Basic Science Discoveries to Clinical Trials. Circ. Res..

[28] Sultan Alvi S., Ansari I.A., Khan I., Iqbal J., Khan M.S. (2017). Potential Role of Lycopene in Targeting Proprotein Convertase Subtilisin/Kexin Type-9 to Combat Hypercholesterolemia. Free Radic. Biol. Med..

[29] Waiz M., Sultan Alvi S., Salman Khan M., Professor A. (2022). Potential dual inhibitors of PCSK-9 and HMG-R from natural sources in cardiovascular risk management. EXCLI J..

[30] Kwon H.J., Lagace T.A., McNutt M.C., Horton J.D., Deisenhofer J. (2008). Molecular Basis for LDL Receptor Recognition by PCSK9. Proc. Natl. Acad. Sci. USA.

[31] Lagace T.A. (2014). PCSK9 and LDLR Degradation: Regulatory Mechanisms in Circulation and in Cells. Curr. Opin. Lipidol..

[32] Lambert G., Sjouke B., Choque B., Kastelein J.J.P., Hovingh G.K. (2012). The PCSK9 Decade: Thematic Review Series: New Lipid and Lipoprotein Targets for the Treatment of Cardiometabolic Diseases. J. Lipid Res..

[33] Ni Y.G., Di Marco S., Condra J.H., Peterson L.B., Wang W., Wang F., Pandit S., Hammond H.A., Rosa R., Cummings R.T. (2011). A PCSK9-Binding Antibody That Structurally Mimics the EGF(A) Domain of LDL-Receptor Reduces LDL Cholesterol in Vivo. J. Lipid Res..

[34] Watkins A.M., Arora P.S. (2014). Anatomy of β-Strands at Protein-Protein Interfaces. ACS Chem. Biol..

[35] Kontoyianni M., McClellan L.M., Sokol G.S. (2004). Evaluation of Docking Performance: Comparative Data on Docking Algorithms. J. Med. Chem..

[36] Petrilli W.L., Adam G.C., Erdmann R.S., Abeywickrema P., Agnani V., Ai X., Baysarowich J., Byrne N., Caldwell J.P., Chang W. (2020). From Screening to Targeted Degradation: Strategies for the Discovery and Optimization of Small Molecule Ligands for PCSK9. Cell Chem. Biol..

[37] Friesner R.A., Murphy R.B., Repasky M.P., Frye L.L., Greenwood J.R., Halgren T.A., Sanschagrin P.C., Mainz D.T. (2006). Extra Precision Glide: Docking and Scoring Incorporating a Model of Hydrophobic Enclosure for Protein-Ligand Complexes. J. Med. Chem..

[38] Lokwani D., Azad R., Sarkate A., Reddanna P., Shinde D. (2015). Structure Based Library Design (SBLD) for New 1,4-Dihydropyrimidine Scaffold as Simultaneous COX-1/COX-2 and 5-LOX Inhibitors. Bioorg. Med. Chem..

[39] Yang C., Chen E.A., Zhang Y. (2022). Protein–Ligand Docking in the Machine-Learning Era. Molecules.

[40] Koes D.R., Baumgartner M.P., Camacho C.J. (2013). Lessons Learned in Empirical Scoring with Smina from the CSAR 2011 Benchmarking Exercise. J. Chem. Inf. Model.

[41] Ragoza M., Hochuli J., Idrobo E., Sunseri J., Koes D.R. (2017). Protein-Ligand Scoring with Convolutional Neural Networks Graphical Abstract HHS Public Access. J. Chem. Inf. Model.

[42] Ivanova L., Tammiku-Taul J., García-Sosa A.T., Sidorova Y., Saarma M., Karelson M. (2018). Molecular Dynamics Simulations of the Interactions between Glial Cell Line-Derived Neurotrophic Factor Family Receptor GFRα1 and Small-Molecule Ligands. ACS Omega.

[43] Genheden S., Ryde U. (2015). The MM/PBSA and MM/GBSA Methods to Estimate Ligand-Binding Affinities. Expert Opin Drug Discov..

[44] Zhu H., Zhang Y., Li W., Huang N. (2022). A Comprehensive Survey of Prospective Structure-Based Virtual Screening for Early Drug Discovery in the Past Fifteen Years. Int. J. Mol. Sci..

[45] Nitulescu M., Alves de Oliveira T., Pires da Silva M., Habib Bechelane Maia E., Marques da Silva A., Gutterres Taranto A. (2023). Virtual Screening Algorithms in Drug Discovery: A Review Focused on Machine and Deep Learning Methods. Drugs Drug Candidates.

